# Decreasing Hepatitis C Virus Infection in Thailand in the Past Decade: Evidence from the 2014 National Survey

**DOI:** 10.1371/journal.pone.0149362

**Published:** 2016-02-12

**Authors:** Rujipat Wasitthankasem, Nawarat Posuwan, Preeyaporn Vichaiwattana, Apiradee Theamboonlers, Sirapa Klinfueng, Viboonsak Vuthitanachot, Napha Thanetkongtong, Siriporn Saelao, Monthana Foonoi, Apinya Fakthongyoo, Jamorn Makaroon, Klaita Srisingh, Duangporn Asawarachun, Somchai Owatanapanich, Norra Wutthiratkowit, Kraisorn Tohtubtiang, Pornsak Yoocharoen, Sompong Vongpunsawad, Yong Poovorawan

**Affiliations:** 1 Center of Excellence in Clinical Virology, Faculty of Medicine, Chulalongkorn University, Bangkok, Thailand; 2 Chumpare Hospital, Chum Phae, Khon Kaen, Thailand; 3 Uttaradit Hospital, Mueang, Uttaradit, Thailand; 4 Lablae Hospital, Lablae, Uttaradit, Thailand; 5 Naresuan University Hospital, Mueang, Phitsanulok, Thailand; 6 Phra Nakhon Si Ayutthaya Hospital, Phra Nakhon Si Ayutthaya, Thailand; 7 King Narai Hospital, Khao Sam Yot, Lop Buri, Thailand; 8 Narathiwat Ratchanakarin Hospital, Mueang, Narathiwat, Thailand; 9 Trang Hospital, Mueang, Trang, Thailand; 10 Bureau of General Communicable Diseases, Department of Disease Control, Ministry of Public Health, Nonthaburi, Thailand; University of Malaya, MALAYSIA

## Abstract

Hepatitis C virus (HCV) infection affects ≥ 180 million individuals worldwide especially those living in developing countries. Recent advances in direct-acting therapeutics promise effective treatments for chronic HCV carriers, but only if the affected individuals are identified. Good treatment coverage therefore requires accurate epidemiological data on HCV infection. In 2014, we determined the current prevalence of HCV in Thailand to assess whether over the past decade the significant number of chronic carriers had changed. In total, 5964 serum samples from Thai residents between 6 months and 71 years of age were obtained from 7 provinces representing all 4 geographical regions of Thailand and screened for the anti-HCV antibody. Positive samples were further analyzed using RT-PCR, sequencing, and phylogenetic analysis to identify the prevailing HCV genotypes. We found that 56 (0.94%) samples tested positive for anti-HCV antibody (mean age = 36.6±17.6 years), while HCV RNA of the core and NS5B subgenomic regions was detected in 23 (41%) and 19 (34%) of the samples, respectively. The seropositive rates appeared to increase with age and peaked in individuals 41–50 years old. These results suggested that approximately 759,000 individuals are currently anti-HCV-positive and that 357,000 individuals have viremic HCV infection. These numbers represent a significant decline in the prevalence of HCV infection. Interestingly, the frequency of genotype 6 variants increased from 8.9% to 34.8%, while the prevalence of genotype 1b declined from 27% to 13%. These most recent comprehensive estimates of HCV burden in Thailand are valuable towards evidence-based treatment coverage for specific population groups, appropriate allocation of resources, and improvement in the national public health policy.

## Introduction

Hepatitis C virus (HCV) infection represents a major public health problem in many countries. Approximately 185 million people are chronically infected and 500,000 people die from HCV-related liver diseases annually [1–2]. As many as 75% of acute infection leads to chronic infection in some individuals, who are often unaware of their HCV status until the appearance of clinical symptoms years later. HCV infection results in liver fibrosis, cirrhosis, and hepatocellular carcinoma, which subsequently requires liver transplantation. Moreover, HCV carriers may unknowingly infect others via blood transfusion and from iatrogenic procedures or intravenous drug use [3].

The standard of care for chronic HCV has been pegylated interferon-alpha administered in combination with ribavirin (PEG-IFN plus ribavirin). However, the treatment does not lead to sustained virologic response (SVR) in all patients due to several factors including the viral genotype, patient age and genetic background, or poor adherence to therapy resulting from adverse events [4–6]. Fortunately, novel and effective direct-acting antivirals (DAA) available in several combination regimens have resulted in > 90% SVR rate in patients with HCV genotype 1, which is typically refractory to standard treatments [7]. Patients with HCV genotypes 2 and 3 who received sofosbuvir plus oral ribavirin treatment can also expect to achieve > 93–95% SVR. Although sofosbuvir treatment is considered cost-effective in developed countries, its use remains cost-prohibitive in developing countries [8–10].

In Thailand, the estimates of HCV prevalence differ considerably depending on the population size, target group, and period of study. The National Blood Center (NBC) has reported that the prevalence of new anti-HCV-positive blood donors declined from 1.6% to 0.5% between 1991 and 2009 [11]. Even though the frequency of HCV among blood donors appears relatively low, it may not reflect the true prevalence in the general population because preliminary screening of blood donors would exclude high-risk individuals such as sex workers, prisoners, intravenous drug users (IVDUs), and blood transfusion recipients [11]. Studies on the general population had reported a decrease in HCV infection from 1.95% in 1994 to 0.86% in 2002 [12–13], but a national survey suggested a higher prevalence of 2.15% in 2004 [14]. Since then, no new data regarding HCV infection in large population-based cohorts are available to indicate more recent national prevalence in the numbers of active and past HCV infection in Thailand. Therefore, the aims of this study are to evaluate the national population-based prevalence of HCV infection and HCV genotype distribution in 2014, and to compare these data to the previous national serosurvey performed in 2004 [14]. These results were then used to estimate the number of viremic carriers and past infection in different age groups.

## Material and Methods

As part of the overall research consortium to assess the status of viral hepatitis in the country (“The impact of hepatitis B vaccine immunization program as part of EPI after 20 years implementation and seroprevalence of hepatitis A, B and C in Thailand”), we determined the prevalence of HCV in the Thai population between January and December 2014. This study was approved by the institutional review board (IRB 419/56) of the Faculty of Medicine, Chulalongkorn University, and in compliance with the principles of the Declaration of Helsinki. Written informed consents were obtained from all participants or their parents (if they were minors).

### Sample collection

**Fig 1:**
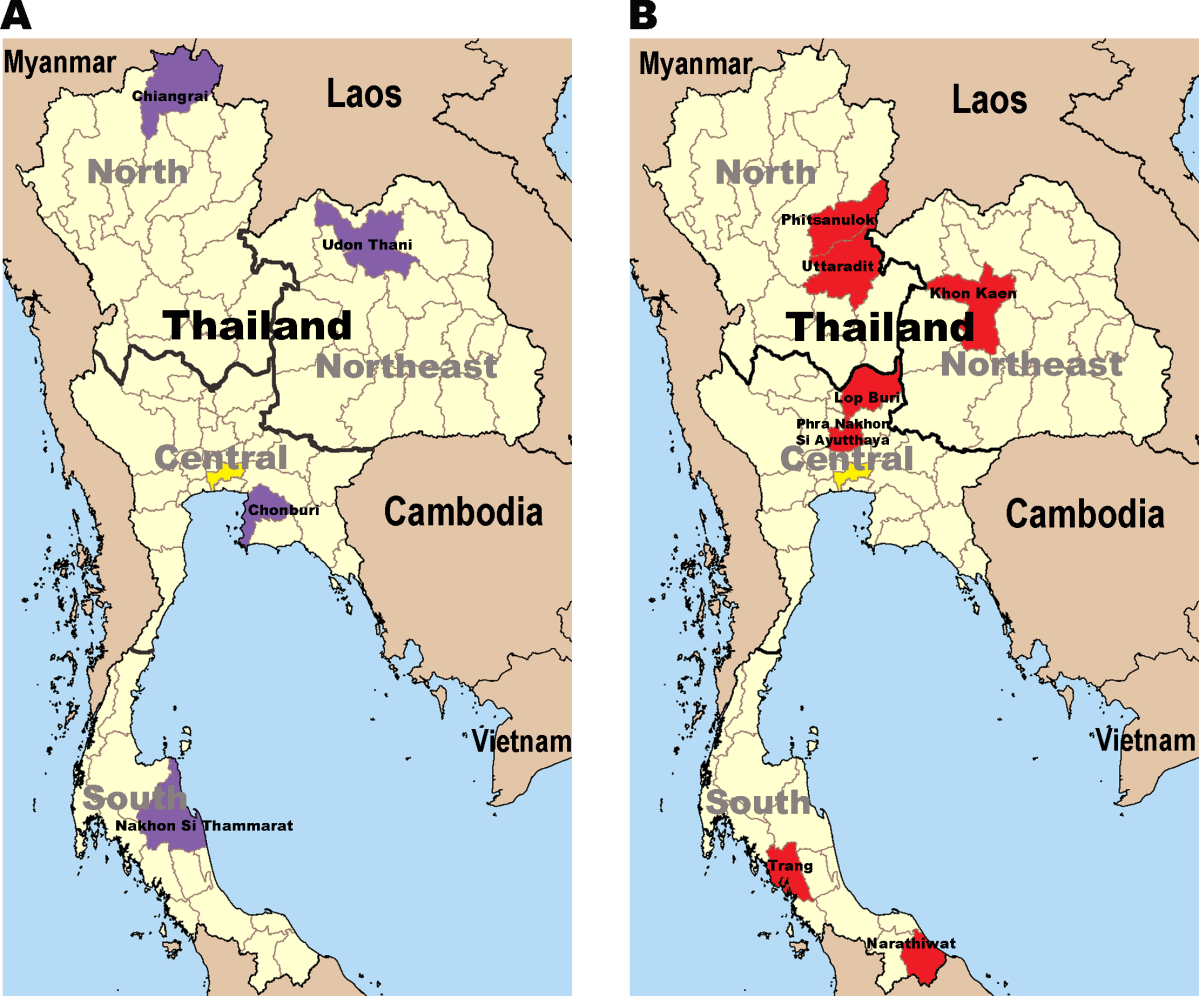
Provinces in Thailand for which HCV seroprevalence was determined. (A) The provinces sampled in 2004 [14] (purple) and (B) in this study (red). The capital city Bangkok is shown in yellow.

A total of 5964 serum samples were collected in 7 provinces (Pha Nakhon Si Ayutthaya, n = 757; Lop Buri, n = 778, Uttaradit, n = 903; Phitsanulok, n = 518; Khon Kaen, n = 1633; Trang, n = 730; and Narathiwat, n = 645) located in 4 different geographical regions of Thailand (north, northeast, central, and south) (Fig 1 and S1 Table). Blood samples were collected from individuals during scheduled pediatric health check-up or outpatient clinic at the hospital. The inclusion criteria were: age between 6 months and 71 years, no history of chronic diseases, no clinical signs or symptoms associated with either an immunodeficiency disorder or HIV infection, and no history of immunosuppressive therapy. The sera from clotted blood samples were collected within 24 hours and kept at -70°C until testing.

### Serological testing

All sera were tested for the HCV antibody using an automated chemiluminescent microparticle immunoassay with recombinant proteins HCr43 (core and NS3) and c100-3 (NS4a and NS4b) (ARCHITECT anti-HCV assay, Abbott Diagnostics, Wiesbaden, Germany) according to the manufacturer’s instructions. Samples identified as HCV antibody-positive by the automated assay were evaluated for their signal-to-cut-off (S/CO) ratio. True HCV antibody-positive result was defined by a high S/CO ratio ≥ 5.0 (Abbott, Architech anti-HCV), while sample with an S/CO ratio < 5.0 was further evaluated according to the U.S. Centers for Disease Control and Prevention (CDC) (http://www.cdc.gov/hepatitis/hcv/labtesting.htm). All HCV antibody-positive samples were subjected to further laboratory testing.

### RT-PCR amplification and phylogenetic analysis

Viral RNA was extracted manually from HCV antibody-positive serum using the guanidinium thiocyanate extraction method. Reverse transcription was performed using random hexamers and ImProm-II Reverse Transcriptase (Promega, Madison, WI, USA). The HCV core and NS5B partial sequences were amplified by polymerase chain reaction (PCR) with the 2X Perfect Tag Plus Master Mix (5 PRIME, Gaithersburg, MD, USA) as previously described [15]. PCR amplicons were sequenced (First BASE Laboratories, Selangor, Malaysia) and analyzed using Chromas LITE (v2.01), BioEdit (v.5.0.9; Ibis Therapeutics, Carlsbad, CA, USA), SeqManPro (DNASTAR, Madison, WI, USA), and BLASTN search (http://www.ncbi.nlm.nih.gov).

The core and NS5B sequence alignments were done using ClustalX v.2.1. After trimming, the alignments (299 nucleotides for the core and 300 nucleotides for NS5B) were subjected to phylogenetic reconstruction using neighbor-joining method and Kimura’s two parameters model implemented in MEGA 6.0 [16]. Bootstrap testing relied on 1,000 replicates. The genotype of each sample was assigned according to where it clustered with the reference strain. The reference subtype sequences used in this study (and accession numbers) were: 1a (M62321, M67463), 1b (D90208, M58335), 1c (D14853), 2a (AB047639, D00944), 2b (D10988, AB030907), 2c (D50409), 3a (D17763, D28917), 3b (D49374), 4a (Y11604), 5a (Y13184, AF064490), 6a (Y12083, AY859526), 6b (D84262), 6c (EF424629), 6d (D84263), 6e (DQ314805), 6f (DQ835760), 6g (D63822), 6h (D84265), 6i (DQ835770), 6j (DQ835769), 6 (D84264), 6l (EF424628), 6m (DQ835767), 6n (DQ278894, DQ835768), 6o (EF424627), 6p (EF424626), 6q (EF424625), 6r (EU408328), 6s (EU408329), 6t (EF632071, EU246939), 6u (EU246940), 6v (EU158186, EU798760), 6w (DQ278892, EU643834), 6xa (EU408330, EU408332), 6xb (JX183552, KJ567645), 6xc (KJ567650, KJ567649), 6xd (KM252791, KM252789) and 6xe (JX183557, KM252792). All nucleotide sequences generated were submitted to the GenBank database under accession numbers KT921354-KT921395.

### Comparison with the previous national survey

Data from this study were compared to those reported by a previously published national survey conducted in 2004 [14]. That study included the demographic data of healthy individuals and those with acute illness (age range = 6 months–60 years) who resided in 4 provinces (Chiang Rai, Udon Thani, Nakhon Si Thammarat, and Chon Buri) representing each geographical region (Fig 1A). In the previous survey, the anti-HCV antibody screening also utilized an automated ELISA assay (AxSYM; Abbott Laboratory, North Chicago, IL, USA). Viral RNA was also isolated and amplified using similar protocols as this study.

The Thai population data in 2004 and 2014 were derived from the Official Statistics Registration Systems (http://stat.dopa.go.th/stat/statnew/upstat_age.php). From these data, estimates of the numbers of both the anti-HCV-positive individuals and viremic carriers were extrapolated to yield the HCV-seropositive and viremic rate in this and the previous study [14]. The number of HCV carriers was calculated according to the population of each age group (0–10, 11–20, 21–30, 31–40, 41–50, and > 50 years), which in total provided the number of carriers in Thailand. The number of anti-HCV-positive individuals, viremic carriers, and genotypic distribution were compared over this 10-year period.

### Data analysis

The Chi-square and Mann-Whitney U test were used to compare the categorical and continuous variables, respectively. In this study, *p* < 0.05 was considered statistically significant. All statistical analyzes were performed using SPSS for Windows (version 11.5; SPSS, Chicago, IL, USA).

## Results

### HCV seroprevalence

The demographic data of all 5,964 individuals included in this study are shown in Table 1. The mean age was 24.55 ± 18.52 years and there were more females than males (male = 2,530 samples, female = 3,434 samples). A total of 56 samples tested positive for anti-HCV antibody (mean age = 36.46 ± 17.16 years). HCV seropositivity was higher in men than women and was highest in the northeastern region (20 positive samples, or 1.22%) and lowest in the southern region (8 positive samples, or 0.58%). There were no significant differences between genders and HCV antibody-positive status regionally (*p* = 0.340 and 0.081, respectively). However, there were statistically significant differences in the mean ages of seropositive individuals in these 4 regions (*p* = 0.044) because the participants in the northeastern region were the oldest (41.30 ± 11.67 years) and those in the southern region were the youngest (20.75 ± 17.17 years).

**Table 1:** Demographic data of individuals in this study.

Region	Central	North	Northeast	South	Total	*p*
**No. of samples**	1535	1421	1633	1375	5964	NA^a^
**Age (SD)**	24.12 18.45)	25.14 (18.63)	23.16 (18.13)	26.08 (18.81)	24.55 (18.52)	NA^a^
**Sex (M/F)**	701/834	580/841	782/851	467/908	2530/3434	NA^a^
**Anti-HCV (%)**	15 (0.98)	13 (0.91)	20 (1.22)	8 (0.58)	56 (0.94)	0.340
** Age (SD)**	40.20 (17.02)	34.38 (19.97)	41.30 (11.67)	20.75 (17.17)	36.46 (17.16)	0.044
** Sex (M/F)**	7/8	8/5	15/5	5/3	35/21	0.081
** HCV RNA +ve(%)**	4 (26.67)	3 (23.08)	14 (70.00)	2 (25.00)	23 (41.07)	NA^a^
** Genotype**						NA^a^
** 1a**	-	-	1	-	1 (4.35)	
** 1b**	-	2	1	-	3 (13.04)	
** 3a**	1	1	6	2	10 (43.48)	
** 3b**	1	-	-	-	1 (4.35)	
** 6c**	-	-	1	-	1 (4.35)	
** 6f**	-	-	3	-	3 (13.04)	
** 6i**	-	-	2	-	2 (8.70)	
** 6j**	1	-	-	-	1 (4.35)	
** 6n**	1	-	-	-	1 (4.35)	

^a^This parameter was not analyzed.

**Fig 2:**
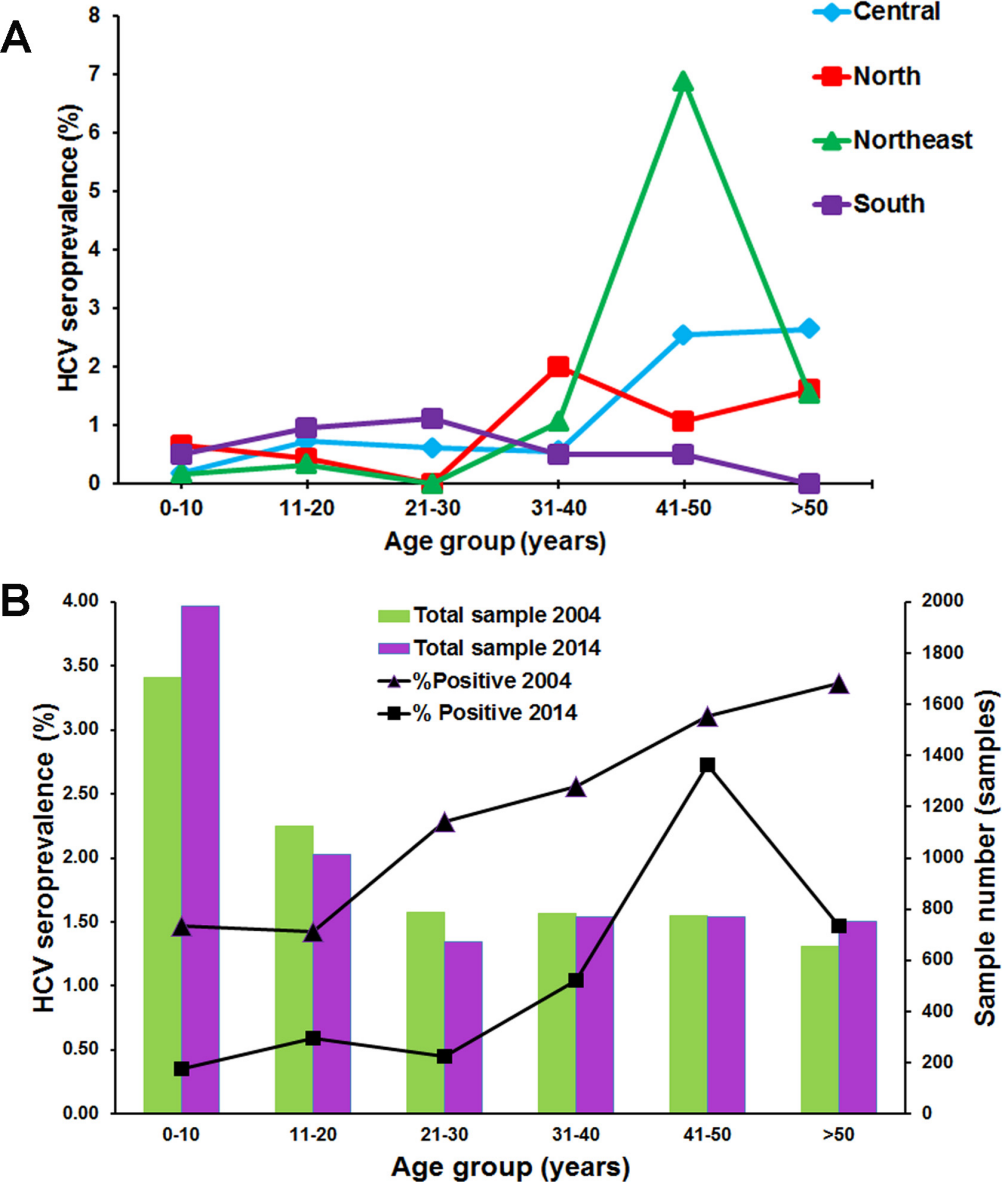
Distribution of HCV seropositive rates in 2014 compared to 2004. (A) The prevalence of anti-HCV antibody was stratified by age group and geographical region. (B) The number of samples examined in 2004 (green) and 2014 (purple) for each age group. The seroprevalence in 2004 (triangle) [14] and 2014 (square) for each age group.

The anti-HCV prevalence was relatively low among individuals < 30 years of age regardless of the region in which they reside (Fig 2A, S1 Table). Regionally, the highest anti-HCV prevalence among individuals 31–40 years was in the north (2.00%), while among the age group 41–50 years was in the northeast (6.88%). Among individuals > 50 years of age, the highest prevalence was in the central region (2.65%). In the south, prevalence of anti-HCV antibody appeared to increase up to the age of 30, then declined among individuals > 30 years.

Given comparable number of samples surveyed in 2004 and 2014, it was noted that the overall seroprevalence in 2004 correlated with increasing age (Fig 2B). In 2014, however, the prevalence of anti-HCV antibody markedly decreased. The seroprevalence was highest among individuals 41–50 years of age (2.72%) and appeared to decline among those > 50 years of age (1.46%).

### HCV viremia

**Fig 3:**
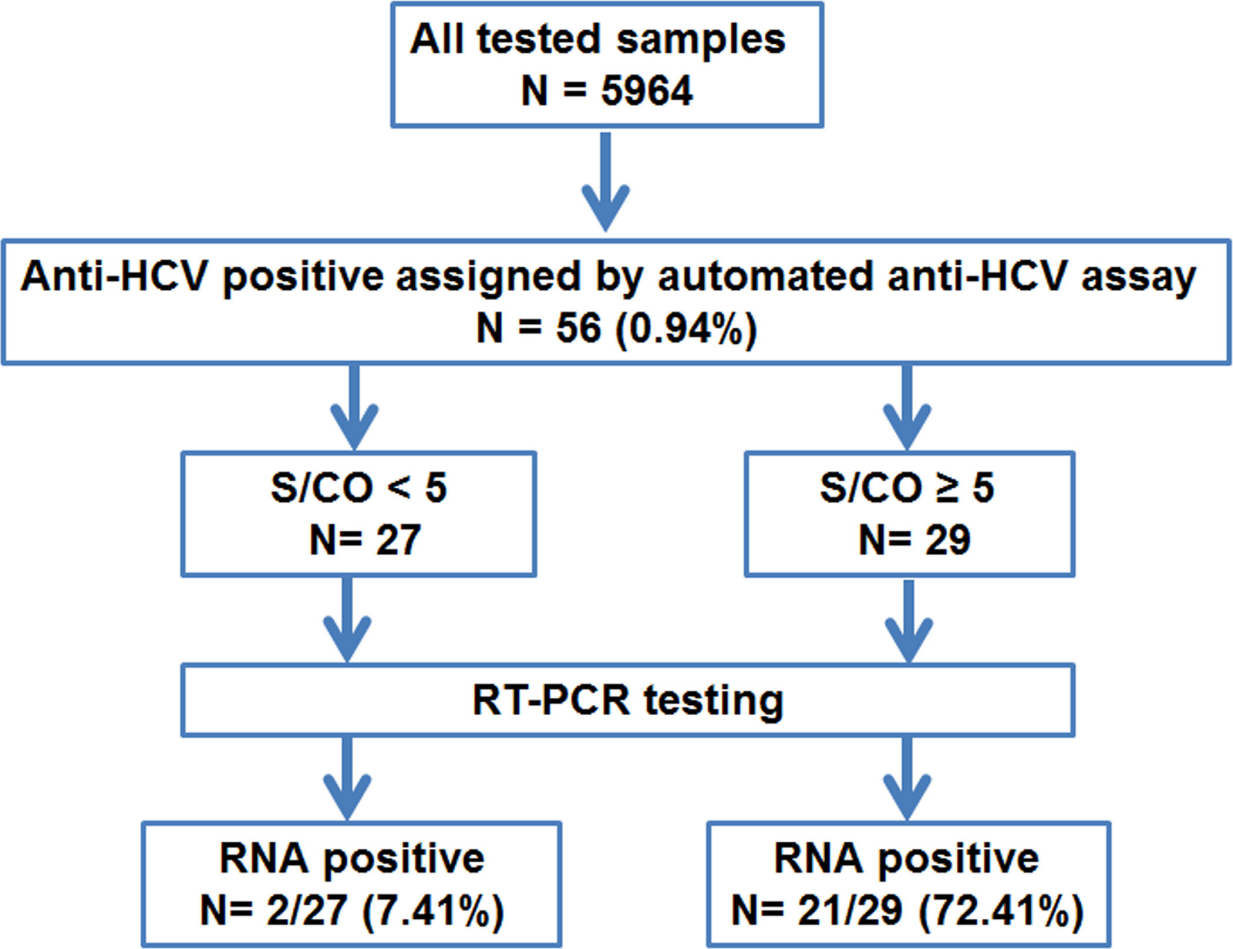
Schematic diagram of the study protocol. S/CO denotes the signal-to-cutoff ratio.

To determine the prevalence of viremia among the 56 seropositive samples, HCV RNA was assessed using RT-PCR analysis of the subgenomic core region. According to the U.S. CDC guidelines of (http://www.cdc.gov/hepatitis/hcv/labtesting.htm), the S/CO ratio for optical density (OD) predictive of the true HCV positivity in > 95% of samples is ≥ 5.0. Therefore, the 56 anti-HCV-positive samples were divided into the low and high S/CO-ratio groups (Fig 3 and S2 Table). For the low S/CO-ratio group (OD < 5), there were 27 samples with a mean OD of 2.34 ± 0.95. Only 2 samples tested positive for viral RNA, giving a viremic rate of 7.41%. Meanwhile, the high S/CO-ratio group (OD ≥ 5.0) comprised 29 samples with a mean OD of 12.39 ± 2.20. However, only 21 samples tested positive for viral RNA and thus yielded a viremic rate of 72.41%. In all, 23 out of 56 seropositive samples (41.07%) tested positive for the HCV core region. We also determined the HCV RNA of the NS5B region, but only 19 samples tested positive (S2 Table). Among the 4 NS5B-negative samples, 2 had low S/CO ratios and 2 others had high S/CO ratios.

### HCV genotypes in Thailand

**Fig 4:**
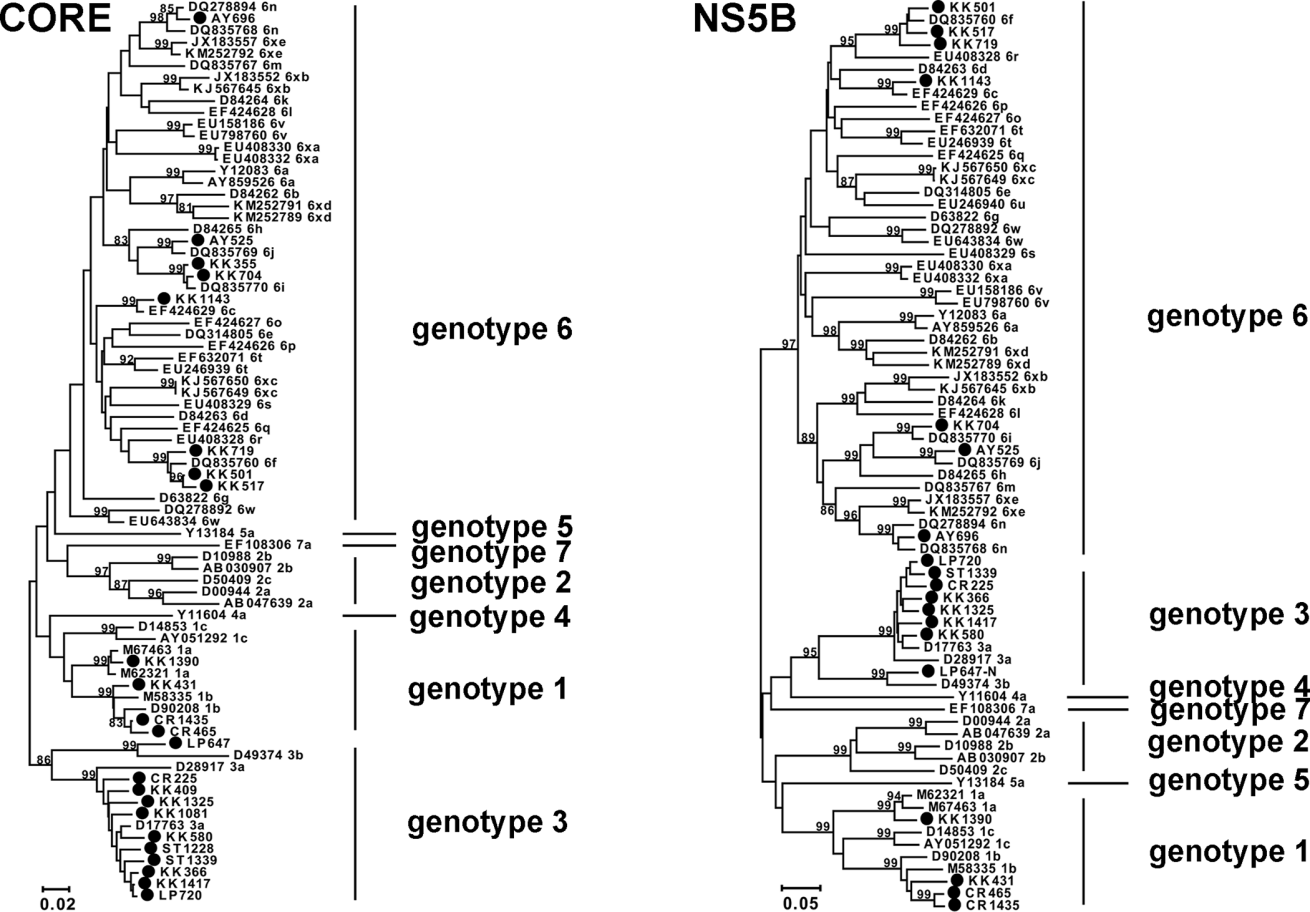
Phylogenetic tree based on the core (left) and NS5B (right) sequences of the HCV-positive samples. The positive samples in this study are indicated (black circles). The accession numbers with the respective genotypes and > 80% bootstrap values are shown.

Phylogenetic analysis of the core and the NS5B regions were consistent in identifying HCV genotypes among the positive samples (Fig 4). The most common HCV belonged to genotype 3a (43.48%), followed by 1b (13.04%), 6f (13.04%), 6i (8.70%), 1a (4.35%), 3b (4.35%), 6c (4.35%), 6j (4.35%), and 6n (4.35%) (Table 1).

### HCV prevalence over time

**Fig 5:**
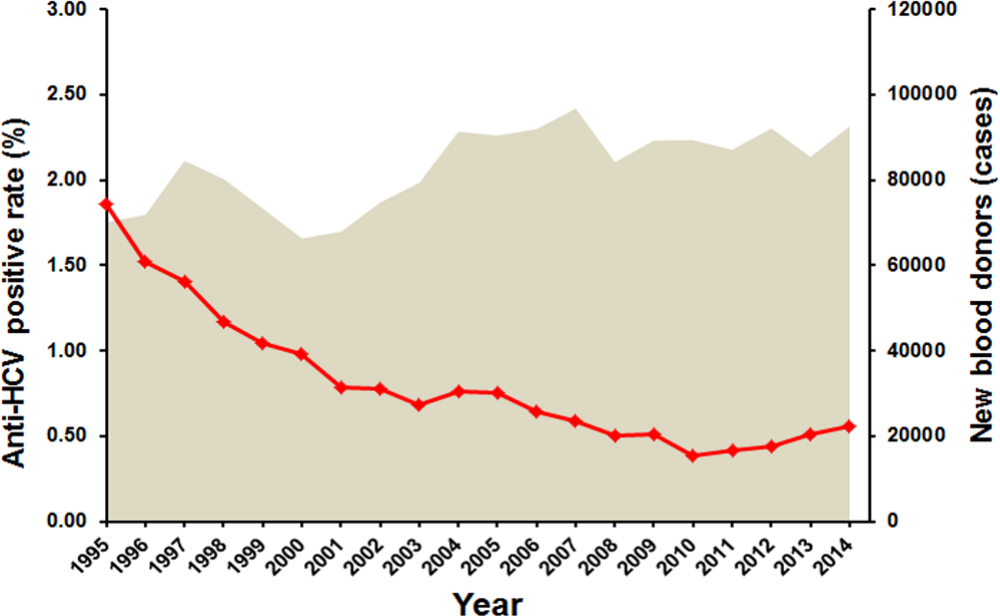
HCV seropositivity rate derived from blood screening by the National Blood Center (NBC) between 1995–2014. The shaded area represents the number of annual screenings of new blood donors performed by NBC. The seroprevalence trend is denoted by the line.

To assess the current trend in HCV infection, we calculated the approximate HCV seroprevalence and viremia in the general population based on the data from this and a previous study (Fig 2B and Table 2) [14]. Comparison of the HCV antibody-positive and RNA-positive rates between 2004 and 2014 showed that the HCV prevalence gradually declined in Thailand. In 2004, approximately 1.4 million people were HCV antibody-positive, and 715,930 people were presumed viremic with appreciable HCV RNA. In 2014, however, there were 758,940 HCV antibody-positive individuals, 356,670 of whom were viremic for HCV. Overall, both HCV seroprevalence and the viremic rate decreased from 2.15% to 0.94% and from 1% to 0.39%, respectively. This trend was in agreement with the seroprevalence screening donated blood screened by the Thailand National Blood Bank (Fig 5). Therefore, the estimated number of HCV carriers decreased by half from 715,930 people in 2004 to 356,670 people in 2014 (Table 2).

**Table 2:** Estimated HCV carriers in the Thai population.

	Year 2004					Year 2014				
Age range	Anti-HCV +ve rate	RNA +ve rate	Thai population	Anti-HCV carrier	HCV carrier	Anti-HCV +ve rate	RNA +ve rate	Thai Population	Anti-HCV carrier	HCV carrier
**0–10**	1.47	0.41	9,553,008	140429	39,244	0.35	0.05	8,485,974	29701	4,273
**11–20**	1.42	0.53	9,419,566	133758	50,238	0.59	0.10	8,829,060	52091	8,699
**21–30**	2.28	1.02	10,570,790	241014	107,318	0.45	0.00	9,330,783	41989	0
**31–40**	2.56	1.92	10,972,787	280903	210,745	1.04	0.13	10,346,437	107603	13,472
**41–50**	3.10	1.55	8,859,873	274656	137,540	2.72	1.69	10,465,811	285061	176,466
**>50**	3.36	1.53	11,173,300	375423	170,846	1.46	0.93	16,496,285	242495	153,760
**Total**	**2.15**	**1.00**	**60,549,324**	**1,446,183**	**715,930**	**0.94**	**0.39**	**63,954,350**	**758,940**	**356,670**

## Discussion

HCV genotype 1a, 1b, 3a, 3b, and 6 variants were identified in this study, which suggested that genotypic distribution remained relatively unchanged over the past decade. HCV genotype 3a continued to be the most common genotype in Thailand (51.5% in the previous study vs 43.5% in the current study). Interestingly, genotype 6 variants significantly increased from 8.9% to 33.8% between 2004 and 2014, while genotype 1b declined by half (from 26.7% to 13.1%). An increase in the distribution of the genotype 6 variants was also observed in the blood donors in some regions of Thailand and IVDUs in China [17–18]. These changes may be associated with behaviors that are transmission route-specific as it has been suggested that the transmission patterns of genotype 1b and 6 were linked to blood transfusion and sharing of contaminated needles or IVDUs, respectively [18–20].

To exclude false-positive anti-HCV results and minimize the number of samples required for additional tests, we used the U.S. CDC guideline to classify HCV antibody-positive samples into 2 groups based on their OD values. As expected, the high S/CO-ratio group in this study demonstrated 72% HCV-RNA positivity upon confirmatory RT-PCR testing, which was in agreement with a previous study examining the anti-HCV-positive antibodies among blood donors (71%) [21]. The higher OD value from the S/CO ratio also correlated with the likelihood of an active HCV infection. In contrast, a lower S/CO ratio was predictive of HCV RNA negativity [22] as HCV RNA was only detected in 2 samples in the low S/CO-ratio group (7%). The latter may be due to several reasons including spontaneous clearance of previous HCV exposure, low viral load, or occult infection [23–24]. HCV viremic individuals with detectable viral RNA ideally would require long-term follow-up to confirm infection in this population. In addition, a more sensitive test may be needed to detect extremely low viral RNA load in some HCV viremic individuals.

Epidemiological trends of HCV infection in Thailand have been difficult to evaluate for several reasons. Sampling differed in locations and consequently showed different rates of infection. For example, a study by a northern regional blood center reported an HCV-positivity rate of 2.16–2.91%, while another blood center in the northeast reported a higher prevalence at 5.62% [25–27]. In addition, the time period of the study also led to different rates of HCV positivity. In 1994, a survey in Bangkok found an anti-HCV-positive incidence of 1.95% in the general population, which was consistent with the 1.86% positivity rate found in blood donors by the National Blood Center (NBC) in Bangkok the very same year [11–12]. However, the rate appeared to decrease to 0.86% according to a study conducted in 2000–2002, which surveyed only individuals residing in the central region of Thailand including Bangkok [13]. The selection of study cohort also influenced HCV prevalence. HCV infection tends to be more frequent among high-risk groups such as IVDUs, hemodialysis patients, and in populations residing in remote areas compared to the general population [28–29]. In particular, HCV is highly prevalent among drug injectors (up to 86%) compared to the general population (approximately 2%) [14,30–31].

Accurately derived HCV seropositive rate ideally requires data analysis with comparable parameters. A similar population-based survey in 2004 by our group suggested that HCV seroprevalence was approximately 2.15%. This was 3 times higher than the 0.76% positivity rate reported by NBC [11,14]. Therefore, this study attempted to include different geographical locations in order to represent the overall population and produce the current HCV rate. Using a comparative sample size, nearly 6,000 individuals were sampled and screened using similar methods as in 2004. Consequently, we found that the HCV prevalence (0.94%) decreased by half compared to a decade ago. The rate was low among younger Thais and slightly higher in older individuals. The increasing HCV prevalence with age in Thailand was also similarly observed in U.S. and Spain [32–33]. The relatively high HCV positive rate in the northeastern region (7.0% in individuals between 41–50 years of age) was concordant with a previous study conducted in 1995 on blood donors in the same region. At that time, a very high prevalence (6.5%) was found in individuals between 21–30 years of age [27]. It is unclear why HCV infection remained relatively unchanged in the northeast, therefore further studies are likely needed to identify HCV transmission and disease burden in this population.

The estimated 345,670 HCV carriers derived from this study was significantly lower than the previously estimated HCV viremic carriers in Thailand [34]. The decreasing prevalence of HCV over the past decade may have resulted from several factors. The discovery of HIV greatly increased the public health awareness of bloodborne pathogens. After the first case of HIV was reported in Thailand [35], there was a significant improvement in the healthcare system and clinical practice. Emphasis on sterilized medical equipment, use of disposable needles, increased screening of donated blood, and the practice of universal precautions all contributed to the reduction of bloodborne agents including HCV, which shares the similar routes of transmission as HIV. In addition, Thailand had placed increased emphasis on the safety of the blood supply since HCV was discovered in 1989. From 1991, mandated screening of donated blood included the use of molecular biology to detect viral nucleic acid beginning in 2006 [11,36–37]. As a result, the number of HCV antibody-positive blood donors had slowly declined from 2% in the early years of implementation to 0.5% in 2014 (Fig 5). Furthermore, declining HCV infection may be related to increased methamphetamine use as it has replaced intravenous heroin injection during the last 10 years. Finally, increased awareness through health education may have also contributed to the reductions in HCV infection in Thailand.

Information on HCV infection in other Southeast Asian countries is either limited or demonstrates varying prevalence rates [38–39]. Generally high HCV prevalence has been reported in Myanmar (11.6%) and Cambodia (5.8%) [40–41], although they are lower among Myanmarian (1.69%) and Cambodian (2.3%) migrants living in Thailand [42]. Intermediate levels of HCV infection have been reported in Vietnam (1.0–3.3%), Indonesia (2.1%), and the Philippines (2.2%) [38–39,43–44]. HCV prevalence among Laotian migrants living in Australia was approximately 3.2% [45]. The present Thailand HCV prevalence of 0.94% resembles that of more developed countries such as Singapore (0.37%) and Malaysia (0.14%) [46–47].

Effective HCV treatments have begun to reduce the overall incidence of HCV in carriers. Recent changes in the Thai healthcare policy have made universal HCV treatment available to all HCV patients, but access to treatment remains a challenge. In other developing countries, highly effective DAA therapy regimens are often not available due to financial barrier, and the cost-effectiveness of using these promising new drugs remains debatable [8,48].

## Supporting Information

S1 Table The number of tested and positive samples in each region categorized by age group. (DOCX)

S2 Table Samples classified by the S/CO ratio and HCV-RNA and genotype results. The HCV genotype based on the core and NS5B sequences are indicated.
(DOCX)

S3 Table Results of a previous study on HCV prevalence in blood donors and the general population. (DOCX)
