# Correction: Decreasing Hepatitis C Virus Infection in Thailand in the Past Decade: Evidence from the 2014 National Survey

**DOI:** 10.1371/journal.pone.0152451

**Published:** 2016-05-18

**Authors:** Rujipat Wasitthankasem, Nawarat Posuwan, Preeyaporn Vichaiwattana, Apiradee Theamboonlers, Sirapa Klinfueng, Viboonsak Vuthitanachot, Napha Thanetkongtong, Siriporn Saelao, Monthana Foonoi, Apinya Fakthongyoo, Jamorn Makaroon, Klaita Srisingh, Duangporn Asawarachun, Somchai Owatanapanich, Norra Wutthiratkowit, Kraisorn Tohtubtiang, Pornsak Yoocharoen, Sineenart Oota, Sompong Vongpunsawad, Yong Poovorawan

Dr. Sineenart Oota is not included in the author byline. She should be listed as the eighteenth author, and her affiliation is: National Blood Center, Thai Red Cross Society, Bangkok 10330, Thailand. The contributions of this author are as follows: Contributed reagents/materials/analysis tools.
