# Supplementary material for: Decreasing Hepatitis C Virus Infection in Thailand in the Past Decade: Evidence from the 2014 National Survey
Source: PLoS One. 2016 Feb 12;11(2):e0149362. doi: 10.1371/journal.pone.0149362 (PMC4752320; doi:10.1371/journal.pone.0149362)
Supplement: S1 Table — (DOCX) [file pone.0149362.s001.docx]

**S1 Table. Number of tested samples and positive samples in each region categorized by age group.**

|  | **Central** | | **North** | | **Northeast** | | **South** | | **Total** | |
| --- | --- | --- | --- | --- | --- | --- | --- | --- | --- | --- |
| **Age range** | **Sample No.** | **Anti-HCV +ve (%)** | **Sample No.** | **Anti-HCV +ve (%)** | **Sample No.** | **Anti-HCV +ve (%)** | **Sample No.** | **Anti-HCV +ve (%)** | **Sample No.** | **Anti-HCV + ve(%)** |
| **0-10** | 537 | 1(0.19) | 456 | 3(0.66) | 590 | 1(0.17) | 403 | 2(0.50) | 1986 | 7(0.35) |
| **11-20** | 273 | 2(0.73) | 226 | 1(0.44) | 307 | 1(0.33) | 209 | 2(0.96) | 1015 | 6(0.59) |
| **21-30** | 162 | 1(0.62) | 166 | 0(0.00) | 166 | 0(0.00) | 179 | 2(1.12) | 673 | 3(0.45) |
| **31-40** | 178 | 1(0.56) | 200 | 4 (2.00) | 188 | 2(1.06) | 202 | 1(0.50) | 768 | 8(1.04) |
| **41-50** | 196 | 5(2.55) | 187 | 2(1.07) | 189 | 13(6.88) | 199 | 1(0.50) | 771 | 21(2.72) |
| **>50** | 189 | 5(2.65) | 186 | 3(1.61) | 193 | 3(1.55) | 183 | 0(0.00) | 751 | 11(1.46) |
| **Total** | **1535** | **15(0.98)** | **1421** | **13(0.91)** | **1633** | **20(1.22)** | **1375** | **8(0.58)** | **5964** | **56(0.94)** |
