# Supplementary material for: Decreasing Hepatitis C Virus Infection in Thailand in the Past Decade: Evidence from the 2014 National Survey
Source: PLoS One. 2016 Feb 12;11(2):e0149362. doi: 10.1371/journal.pone.0149362 (PMC4752320; doi:10.1371/journal.pone.0149362)
Supplement: S2 Table — The HCV genotype based on the core and NS5B sequences are indicated. (DOCX) [file pone.0149362.s002.docx]

**S2 Table. Samples classified by Signal-to-Cut-Off-Ratio (s/co) with HCV-RNA and genotype results.** HCV genotype based on Core and NS5B sequence were indicated.

| **Low s/co ratio (OD<5)** | | | | | | | **High s/co ratio (OD≥5)** | | | | | | |
| --- | --- | --- | --- | --- | --- | --- | --- | --- | --- | --- | --- | --- | --- |
| **No.** | **Code** | **OD** | **Age** | **Sex** | **Core** | **NS5B** | **No.** | **Code** | **OD** | **Age** | **Sex** | **Core** | **NS5B** |
| **1** | KK0355 | 2.49 | 42 | M | 6i | - | **1** | KK0366 | 9.45 | 51 | F | 3a | 3a |
| **2** | KK0370 | 1.19 | 33 | M | - | - | **2** | KK0409 | 11.3 | 41 | M | 3a | - |
| **3** | KK0533 | 2.03 | 43 | F | - | - | **3** | KK0431 | 12.2 | 42 | M | 1b | 1b |
| **4** | KK0575 | 4.29 | 50 | M | - | - | **4** | KK0501 | 12.77 | 41 | M | 6f | 6f |
| **5** | KK1037 | 1.23 | 7 | F | - | - | **5** | KK0517 | 12.59 | 47 | M | 6f | 6f |
| **6** | LP0282 | 2.65 | 50 | M | - | - | **6** | KK0580 | 13.12 | 48 | M | 3a | 3a |
| **7** | LP0593 | 1.47 | 57 | F | - | - | **7** | KK0704 | 11.25 | 50 | F | 6i | 6i |
| **8** | LP0626 | 3.13 | 19 | M | - | - | **8** | KK0719 | 14.44 | 51 | M | 6f | 6f |
| **9** | LP0630 | 4.03 | 29 | M | - | - | **9** | KK0778 | 12.49 | 53 | F | - | - |
| **10** | LP0675 | 2.87 | 52 | F | - | - | **10** | KK1081 | 13.41 | 14 | M | 3a | - |
| **11** | AY0041 | 1.09 | 1 | F | - | - | **11** | KK1143 | 14.06 | 44 | M | 6c | 6c |
| **12** | AY0490 | 2.1 | 51 | F | - | - | **12** | KK1325 | 12.68 | 45 | F | 3a | 3a |
| **13** | AY0579 | 4.53 | 16 | F | - | - | **13** | KK1390 | 17.42 | 43 | M | 1a | 1a |
| **14** | CR0095 | 2.18 | 35 | M | - | - | **14** | KK1417 | 9.85 | 43 | M | 3a | 3a |
| **15** | CR0531 | 1.07 | 12 | F | - | - | **15** | KK1434 | 11.96 | 38 | M | - | - |
| **16** | CR0924 | 1.7 | 7 | F | - | - | **16** | LP0292 | 13.89 | 42 | F | - | - |
| **17** | CR1165 | 3.07 | 34 | F | - | - | **17** | LP0647 | 11.72 | 42 | M | 3b | 3b |
| **18** | CR1187 | 2.57 | 40 | F | - | - | **18** | LP0690 | 9.76 | 34 | M | - | - |
| **19** | CR1674 | 1.5 | 47 | M | - | - | **19** | LP0720 | 13.47 | 55 | M | 3a | 3a |
| **20** | CR1681 | 1.55 | 47 | M | - | - | **20** | AY0344 | 13.32 | 47 | F | - | - |
| **21** | CR1743 | 1.76 | 7 | M | - | - | **21** | AY0525 | 10.35 | 58 | F | 6j | 6j |
| **22** | ST0033 | 2.54 | 21 | F | - | - | **23** | AY0696 | 13.65 | 50 | M | 6n | 6n |
| **23** | ST0081 | 2.02 | 26 | F | - | - | **23** | CR0101 | 11.44 | 40 | F | - | - |
| **24** | ST0713 | 3.51 | 12 | M | - | - | **24** | CR0225 | 11.21 | 59 | M | 3a | 3a |
| **25** | ST0721 | 2.12 | 1 | M | - | - | **25** | CR0465 | 14.56 | 59 | M | 1b | 1b |
| **26** | ST0995 | 2.5 | 18 | M | - | - | **26** | CR1435 | 13.77 | 54 | M | 1b | 1b |
| **27** | ST1228 | 1.88 | 0 | M | 3a | - | **27** | CR1825 | 5.23 | 6 | M | - | - |
|  |  |  |  |  |  |  | **28** | ST0434 | 14.43 | 49 | M | - | - |
|  |  |  |  |  |  |  | **29** | ST1339 | 13.57 | 39 | F | 3a | 3a |
