# Supplementary material for: Decreasing Hepatitis C Virus Infection in Thailand in the Past Decade: Evidence from the 2014 National Survey
Source: PLoS One. 2016 Feb 12;11(2):e0149362. doi: 10.1371/journal.pone.0149362 (PMC4752320; doi:10.1371/journal.pone.0149362)
Supplement: S3 Table — (DOCX) [file pone.0149362.s003.docx]

**S3 Table. Previous study of HCV prevalence in blood donor and general population.**

| **Region** | **Province** | **Collected year** | **Sample group** | **Sample No.** | **Anti-HCV +ve(%)** | **Commercial test** | **Reference** |
| --- | --- | --- | --- | --- | --- | --- | --- |
| Central | Bangkok | 1993-1994 | General population | 616 | 12(1.95%) | Abbott HCV EIA 2.0 | Suwanagool S, J Med Assoc Thai;1995 |
| North | Not idicated | 1994 | Blood donors | 3053 | 66(2.16%) | In house EIA | Sawanpanyalert P, J Epidemio Commun Health; 1996 |
| Northeast | KhonKane | 1995 | Blood donors | 3255 | 183(5.62%) | Organon Teknika HCV EIA 2.0 and Murex HCV EIA 3.0 | Songsirivilai S, Am.J.Trop.Med;1997 |
| North | Phitsanulok | 1999 | Blood donors | 2167 | 63(2.91%) | Abbott HCV EIA 3.0 | Luksamijarulkul P, Southeast Asia J Trop Med; 2002 |
| North | Mae Hong Sorn and Chiang Rai | 1996-1997 | Hill tribes | 658 | 25(3.80%) | Ortho HCV Ab PA test II | Ishida T, J Clin Viro; 2002 |
| Central | Nakorn Pathom, Kanchanaburi, Petchaburi, Ratchaburi, Prachuab Khiri Khan, Suphan Buri and Bangkok | 2000-2002 | General population | 1514 | 13(0.86%) | Abbott HCV EIA 3.0 | Ratanasuwan W, Southeast Asia J Trop Med; 2002 |
| North | Chiang Mai | 2001-2002 | Blood donors | 38,340 | 529(1.38%) | Abbott HCV EIA 3.0 | Thaikruea L, Transfusion; 2004 |
| Central, North, Northeast and South | Chon Buri, Chiang Rai, Udorn Thani and Nakorn Sri Thammarat | 2004 | General population | 5825 | 125(2.15%) | Abbott AxSYM HCV v. 3.0 | Sunanchaikarn S, Asian Pac J Allergy Immunol; 2007 |
| Central, North, Northeast and South | Pha Nakhon Si Ayutthaya, Lop Buri, Uttaradit, Phitsanulok, Khon Kaen, Trang and Narathiwat | 2014 | General population | 5964 | 56(0.94%) | ARCHITECT Anti-HCV assay | This study |
